# A 5′ Promoter Region SNP in *CTSC* Leads to Increased Hypoxia Tolerance in Changfeng Silver Carp (*Hypophthalmichthys molitrix*)

**DOI:** 10.3390/ani15040532

**Published:** 2025-02-13

**Authors:** Nannan Feng, Xiaohui Li, Hang Sha, Xiangzhong Luo, Guiwei Zou, Jiquan Zhang, Hongwei Liang

**Affiliations:** 1 Laboratory of Zoological Systematics and Application of Hebei Province, College of Life Sciences, Hebei University, Baoding 071002, China; fengnannan@yfi.ac.cn; 2 Yangtze River Fisheries Research Institute, Chinese Academy of Fishery Sciences, Wuhan 430223, China; lixiaohui@yfi.ac.cn (X.L.); sh1812@yfi.ac.cn (H.S.); lxz@yfi.ac.cn (X.L.); zougw@yfi.ac.cn (G.Z.); 3 Hubei Hongshan Laboratory, Huazhong Agricultural University, Wuhan 430070, China

**Keywords:** *Hypophthalmichthys molitrix*, hypoxia, SNP, *CTSC*, HIF-1α

## Abstract

Silver carp is a critically significant species in freshwater aquaculture in China, characterized by its limited tolerance to hypoxia. In this study, a significant SNP locus at Chr8: 29647765 (T/C) associated with hypoxia tolerance traits was identified in Changfeng silver carp, and the homozygotic CC genotype exhibited higher hypoxic tolerance than the homozygotic TT and heterozygotic TC genotypes. Under hypoxic conditions, the hemoglobin concentration increased, with the CC genotype demonstrating a significantly higher level compared with the TT genotype; the activities of antioxidant enzymes including catalase and superoxide dismutase were significantly higher in the CC genotype than in the other genotypes; the area of the gill lamellae was significantly smaller in the CC genotype than in the TT and TC genotypes; and the number of apoptotic cells in the brain was significantly lower in the CC genotype than in the TT and TC genotypes. Sequence analysis showed that this SNP was located in the promoter region of the cathepsin C (CTSC) gene. The expression levels of the *CTSC* gene were analyzed across the three genotypes, revealing that the CC genotype exhibited significantly lower expression compared with the TT and TC genotypes under hypoxia. This finding suggests that the SNP associated with the CC genotype leads to reduced *CTSC* expression, which may facilitate better physiological adaptation to hypoxia. Analysis of the promoter region of *CTSC* found a unique predicted hypoxia-inducible factor 1-alpha (HIF-1α) binding site (CGTG) in the T genotype, implying that the differential expression of *CTSC* among the three genotypes under hypoxic stress may be regulated by HIF-1α, a transcription factor integral to hypoxia adaptation, thereby affecting hypoxia tolerance, which further affects the immune response of the Changfeng silver carp in response to the hypoxic environment. Although SNPs represent significant genetic determinants, their phenotypic effects are predominantly mediated through complex interactions within gene regulatory networks and environmental influences. This study identified an effective SNP site in Changfeng silver carp, providing valuable guidance for future selective breeding and the development of new hypoxia-tolerant varieties.

## Simple Summary

In this study, a critical single nucleotide polymorphism (SNP) locus, Chr8:29647765 (T/C), was identified in Changfeng silver carp, demonstrating a significant association with hypoxia tolerance traits. The CC genotype at this locus exhibited superior hypoxia tolerance relative to the TT and TC genotypes. This enhanced tolerance was evidenced by higher hemoglobin concentration, greater antioxidant enzyme activity, smaller gill lamellae area, and fewer apoptotic brain cells under hypoxia stress. This SNP locus is situated within the promoter region of the *CTSC* gene. Under hypoxic conditions, the expression of *CTSC* in individuals with the CC genotype was observed to be lower compared with other genotypes, which may contribute to adaptation to hypoxia. Furthermore, a binding site for HIF-1α was present in the T genotype, which may affect hypoxia tolerance by regulating *CTSC* expression via HIF-1α. These findings provide important molecular markers and a theoretical basis for the selective breeding of Changfeng silver carp with enhanced hypoxia tolerance.

## 1. Introduction

Dissolved oxygen (DO) is essential for the survival and metabolism of aquatic organisms and plays a crucial role in their development, growth, and energy metabolism [1]. The hypoxic response is a complex process involving multiple physiological and biochemical changes in fish [2], which result in delayed locomotion [3], reduced food consumption [4], strong immune response [5], and reduced fecundity [6]. Under hypoxia, organisms undergo a series of adaptations, and fish undergo compensatory changes in hematological levels to improve the oxygen affinity of hemoglobin molecules and the oxygen-carrying capacity of the blood by releasing stored erythrocytes and accelerating the erythrocyte maturation and erythropoiesis of circulating erythrocytes [7]. In addition, gills are the primary organ of respiration in fish, and many fish can cope with hypoxia by modifying the gill structure to increase the lamellar respiratory surface area [8]. For instance, in crucian carp (*Carassius auratus*), exposure to hypoxic and elevated temperature conditions results in a reduction in cell mass surrounding the gill fillets, consequently increasing the respiratory surface area as an adaptive response to hypoxia [9]. Similarly, grass carp (*Ctenopharyngodon idella*) exhibit an adaptive response to hypoxic conditions by augmenting their respiratory surface area, a process facilitated through the apoptosis of gill filaments [10]. Under normal physiological conditions, organisms maintain a delicate equilibrium in reactive oxygen species (ROS) levels through tightly regulated antioxidant defense systems [11]. However, during hypoxic stress, fish undergo a metabolic shift from aerobic to anaerobic pathways due to insufficient oxygen availability for energy production. This metabolic transition results in excessive ROS generation [12]. The resulting oxidative imbalance can trigger a cascade of detrimental effects including oxidative stress-induced cellular damage, apoptotic pathway activation, and potential immune system dysfunction, ultimately leading to the initiation of inflammatory responses [13,14].

Single nucleotide polymorphisms (SNPs) are DNA sequence polymorphisms caused by variations in a single nucleotide at the genomic level. Since their discovery, SNPs have emerged as the most promising genetic markers. In recent years, genome-wide association analysis (GWAS) has emerged as a pivotal method for elucidating the genetic mechanisms underlying various traits. The fundamental principle of GWAS involves employing logistic or linear regression techniques to identify SNPs associated with the traits of interest. Subsequently, the relationships between these genetic variations and the target traits are validated through related functional studies [15]. Association analysis has been extensively employed to identify SNP markers linked to various traits in aquatic animals. In fish research, two SNP markers have been found to be associated with growth in the Chinese soft-shelled turtle (*Pelodiscus sinensis*) [16], and seven SNPs and related genes for hypoxia tolerance have been identified in the yellow croaker (*Larimichthys crocea*) using genome-wide linkage and transcriptome analyses [17]. SNP, a new marker-assisted breeding technology, can be used for selection at the molecular level to accelerate the breeding process and help breed new fish varieties.

Silver carp (*Hypophthalmichthys molitrix*) is one of the four major fishes in China and is economically important, with a wide natural distribution in freshwater waters across the country and plays a positive role in regulating the ecological environment of the waters [18]. The total aquaculture production of silver carp reached 3,879,800 tons in 2023, ranking second among all freshwater aquaculture species, second only to grass carp (5.9 million tons), with an annual gross output value of more than CNY 30 billion, making its industrial status quite important [19]. Silver carp live in the middle and upper layers of the water body and exhibit poor hypoxia tolerance [20]. Hypoxia-induced mortality results in significant annual losses to the aquaculture industry, adversely impacting the economic returns for farmers. Furthermore, the decomposition process releases toxic substances, such as ammonia and hydrogen sulfide, which contaminate aquatic environments and pose risks to fish and other aquatic organisms. Additionally, the reduction in silver carp populations, which consume plankton, may lead to excessive plankton proliferation and subsequent algal blooms. This exacerbates water body anoxia, thereby contributing to ecological disturbances. Consequently, the identification and application of SNP markers associated with hypoxia tolerance represent a crucial strategy for developing aquaculture species with improved resilience to hypoxic stress. To this end, we conducted a GWAS to screen for SNP loci correlated with the oxygen consumption rate in Changfeng silver carp. This newly developed variety, characterized by its rapid growth rate and exceptional hypoxia tolerance, was established through an integrated breeding approach combining artificial gynogenesis, molecular marker-assisted selection, and systematic population breeding [21].

In this study, we employed the Changfeng silver carp as the focal organism and validated GWAS data previously acquired in our laboratory. Our objective was to identify SNP loci associated with hypoxia tolerance traits. Subsequently, we conducted an in-depth analysis of the dominant genotypes at the identified SNP loci to evaluate their performance under hypoxic conditions. Should such an association be confirmed, these SNP loci could serve as valuable markers in future selective breeding programs targeting the enhancement of hypoxia tolerance in silver carp.

## 2. Materials and Methods

### 2.1. Experimental Fish

The Changfeng silver carp used in this study were from a Changfeng silver carp breeder farm in Xishui City, Hubei Province. A total of 5000 Changfeng silver carp (body weight 14.52 ± 4.3 g, body length 9.5 ± 1.2 cm) from the same pond were randomly selected and transferred to the National Silver Carp Germplasm Resource Farm in Ezhou City, Hubei Province, where they were reared in ponds utilizing dechlorinated recycled tap water. Subsequently, these fish were reared in a pond utilizing dechlorinated recycled tap water. The rearing conditions were maintained at a temperature of 25 ± 0.1 °C and a DO concentration of 7.0 ± 0.5 mg/L for a duration of 7 days.

### 2.2. Screening for SNPs Associated with Hypoxia Tolerance in Changfeng Silver Carp

To establish populations of hypoxia-intolerant and hypoxia-tolerant Changfeng silver carp, a total of 1150 individuals were distributed across 23 tanks, with each tank (dimensions: 51 cm × 38 cm × 29 cm) housing 50 fish. By covering the top of the tank with plastic film, this allowed the DO levels to decrease gradually due to fish respiration. DO values were monitored using a portable multiparameter meter (HQ1130, HACH, Loveland, CO, USA). Loss of equilibrium (LOE) was chosen as an indicator of hypoxia, taking into account physiological and behavioral determinants [14]. As the duration of hypoxia extended, individual fish in each tank began to exhibit a loss of equilibrium. The first five fish to lose balance were designated as the hypoxia-intolerant group, with the average dissolved oxygen concentration recorded at 0.34 mg/L at this point. Subsequently, as additional fish lost balance, the final five fish remaining were classified as the hypoxia-tolerant group, with the average dissolved oxygen concentration measured at 0.24 mg/L at this juncture. In total, 115 Changfeng silver carp were categorized into hypoxia-tolerant and hypoxia-intolerant groups. The caudal fins of the hypoxia-intolerant and hypoxia-tolerant groups were taken separately and placed in anhydrous ethanol for preservation.

In a previous study, we identified 52 SNP loci associated with the oxygen consumption rate (unpublished data); however, their relationship with hypoxic tolerance traits remains unclear. Genomic DNA was extracted from both the hypoxia-tolerant and hypoxia-intolerant groups using a genomic DNA extraction kit (NMG0611-100, Wuhan NAMI Biotechnology Co., Wuhan, Hubei, China) and subsequently subjected to electrophoresis on a 1% agarose gel. Samples deemed acceptable were forwarded to Wuhan Tianyi Huiyuan Biotechnology Co. Ltd. (Wuhan, Hubei, China). for genotyping via the SNaPshot assay. Due to unsuccessful amplification in two samples from the hypoxia-tolerant group, a total of 113 results were obtained. The genotypic frequencies of each SNP locus in the hypoxia-tolerant and non-hypoxia-tolerant groups were analyzed using the chi-square test, and the SNP loci significantly associated with hypoxia-tolerant traits were identified.

### 2.3. Hypoxia Tolerance Tests Across Various Genotypes of the Changfeng Silver Carp

To screen for various genotypes of Changfeng silver carp at significant SNP loci, caudal fins were randomly clipped from 300 specimens. Specific primer sequences for the SNP regions were designed using PrimerPremier 5 (version 5.0, Palo Alto, CA, USA) (see Table S1). Genotyping was conducted through sequencing, and the results were analyzed using SnapGene (version 6.0.2, Chicago, IL, USA). This analysis identified a total of 83 TT genotypes, 165 TC genotypes, and 52 CC genotypes among the Changfeng silver carp.

To assess the hypoxia tolerance of various genotypes of Changfeng silver carp, a total of twenty fish from each of the three genotypes (TT, TC, and CC) were selected for experimentation. These fish were placed in a tank measuring 70 cm × 53 cm × 37 cm, with the three genotypes separated by a perforated divider. The tank was sealed with plastic film to facilitate natural oxygen depletion. A schematic representation of the experimental setup is provided in Figure S1. The time at which a fish lost equilibrium and failed to regain its normal posture within 10 s was recorded as the endpoint.

### 2.4. Hypoxia Treatments and Sample Collection

To elucidate the physiological, biochemical, histological, and molecular-biological characteristics of Changfeng silver carp with the preponderant SNP genotypes, thirty individuals representing three distinct genotypes (TT, TC, and CC) were selected and placed into a circular pool for continuous hypoxia experiments. Hypoxic conditions were achieved by pumping nitrogen directly into the water until the DO reached 2 mg/L. The N_2_ supply was cut off, and slow aeration was used to maintain the DO at 2 ± 0.3 mg/L. The DO values were monitored every 20 min using a portable multiparameter meter and adjusted as needed to maintain hypoxic conditions. Fish were anesthetized with 100 mg/L MS-222 and sampled at 0 h (Hy_0h) and 24 h (Hy_24h) after the start of the experiment, with 15 fish of each genotype, and blood, liver, brain, heart, and gill tissues were collected. Samples for hematoxylin and eosin (H&E) staining and TUNEL staining were fixed in Brinell’s solution (BL539A, PARKnSHOP, Hong Kong, China), tissue samples for the electron microscope observations were fixed in glutaraldehyde solution (BL911A, PARKnSHOP, China), and tissue samples for the enzyme activity assay and RNA extraction were immediately frozen in liquid nitrogen and stored at −80 °C.

### 2.5. Blood Analysis and Enzyme Activity Testing

Blood from experimental fish of the three genotypes, TT, TC, and CC, was drawn from the tail vein of the fish with a heparin-flushed 1 mL plastic syringe, and hemoglobin concentration and red blood cell count were quantified using a hematology analyzer (BC2800VET, Mindray, Shenzhen, Guangdong, China).

Liver tissue samples of the three genotypes were homogenized (1:9, *w*/*v*) in ice-cold phosphate buffer (0.1 M, pH 7.4). Subsequently, the homogenates obtained were centrifuged at 3000× *g* at 4 °C for 10 min. Then, the supernatant was collected and the activities of superoxide dismutase (SOD), catalase (CAT), sodium-potassium pump (Na^+^/K^+^-ATP), malondialdehyde, glycogen, and lactate were measured three times in parallel for each genotype using the corresponding commercial kit (Nanjing Jianjian Bioengineering Institute, Nanjing, China). All enzyme analyses were performed within 12 h of the above treatments. Details of the assay kits used are shown in Table S2.

### 2.6. Gill Morphology Measurements

Three gill tissues preserved in 2.5% glutaraldehyde were selected from each group, and then the samples were rinsed three times with phosphate buffer. Subsequently, they were fixed with 1% osmium tetroxide for 1 h at 4 °C and rinsed with phosphate-buffered saline (PBS). The samples were then subjected to a graded ethanol dehydration series ranging from 50% to 100%, followed by conventional drying after replacement with isoamyl acetate. Finally, the specimens were vacuum ion-coated and observed under a scanning electron microscope (SU8100, Hitachi, Tokyo, Japan) where they were photographed three times in parallel for each genotype.

Three gill tissues preserved in 4% paraformaldehyde selected from each group were fixed for 24 h, dehydrated, then cleaned with xylene and embedded in paraffin. The tissues were sectioned at a thickness of 5 μm, rendered transparent using xylene, and re-embedded in paraffin. Serial sections of 5 μm thickness were obtained, stained with hematoxylin and eosin, and subsequently examined and photographed using a light microscope (NIKON ECLIPS C1, Nikon, Tokyo, Japan) three times in parallel for each genotype.

Gill morphometric parameters were measured using ImageJ software (version 2.35, Bethesda, MD, USA). Measurements were taken in 10 randomly selected fields of view in each group including 10 randomly selected gill vignettes and 10 randomly selected pseudopods of each genotype in 3 replications. Measurements were taken to assess changes in the respiratory area of the gill lamellae and the thickness of the interlayer matrix [22].

### 2.7. TUNEL Staining for Cell Apoptosis in Brain Tissues

For each group, three brain tissues preserved in 4% paraformaldehyde were selected for drying and then washed with xylene, embedded in paraffin, and sectioned to a thickness of 5 μm. Apoptotic cells within the sections were detected using a Terminal deoxynucleotidyl transferase (TdT) dUTP Nick-End Labeling (TUNEL) Assay Kit (G1507-100T, G1501-100T, Servicebio, Wuhan, Hubei, China) according to the manufacturer’s instructions. Following incubation with TdT incubation buffer, the sections were then processed using two methods: (1) incubation with streptavidin-HRP, color development with diaminobenzidine (DAB), counterstaining with hematoxylin, and observation under a light microscope (Pannoramic MIDI, 3DHISTECH, Budapest, Hungary); (2) staining with 4′,6-diamidino-2-phenylindole (DAPI), sealing with an anti-fluorescence quenching sealer, and observation under a fluorescence microscope (DM3000LED, LEICA, Wetzlar, Hesse, Germany) three times in parallel for each genotype. Randomly selected fields of view were measured for cell number using ImageJ software to compare the apoptosis rate (number of apoptotic brain cells/total number of brain cells) in sections of the three different genotypes, with 10 measurements for each genotype.

### 2.8. Expression Characterization of the CTSC Gene

To analyze the expression distribution of the SNP-associated gene *CTSC* in tissues and the characteristic changes in response to hypoxia, 36 Changfeng silver carp were randomly selected without regard to genotype, and the same experimental treatments as in Section 2.4 described above, and samples were taken from the liver, brain, heart, gill, muscle, spleen, and kidney tissues at 0 h (Hy_N0h) of the experiment, and the liver, brain, heart, and gill tissues at 6 h (Hy_N6h), 12 h (Hy_N12h), and 24 h (Hy_N24h). Samples were immediately frozen in liquid nitrogen and stored at −80 °C.

### 2.9. RNA Extraction and Quantitative Real-Time PCR (qRT–PCR)

Three tissue samples from each group were selected for total RNA extraction using TRIzol (Invitrogen, Waltham, MA, USA). Total RNA from each sample was reverse transcribed into cDNA using the PrimeScriptTM RT Reagent Kit (TaKaRa, Kusatsu, Shiga, Japan). The qRT-PCR was performed using the QuantStudioTM 6 Flex Real-Time PCR System (Applied Biosystems, Carlsbad, CA, USA). The relative expression levels of the target genes were calculated using the 2^−ΔΔCT^ method, and the resulting data were subjected to statistical analysis to ascertain their significance. The samples were tested in triplicate, and the gene expression levels were normalized to that of 40 s ribosomal RNA three times in parallel for each genotype.

### 2.10. Promoter Analysis

The genes of 10 kb upstream and downstream of significant SNPs were scanned [23,24], and the location of SNPs in the silver carp genome was used to determine the related genes. The promoter sequence encompassing 2000 base pairs upstream of the start codon of the SNP-associated gene was extracted utilizing TBtools software (version 2.35, Guangzhou, Guangdong, China) [25]. This sequence was subsequently submitted to the BDGP Neural Network Promoter Prediction tool (https://fruitfly.org/seq_tools/promoter.html, accessed on 3 July 2024) for the identification of core promoter elements.

### 2.11. Transcription Factor Binding Site Prediction

The prediction of hypoxia-inducible factor (HIF) binding sites within the promoter region of the SNP-associated gene was conducted utilizing the Jaspar database (https://jaspar.elixir.no, accessed on 5 July 2024), with the relative profile score threshold (RPST) parameter set at 80%.

### 2.12. Data Analysis

One-way analysis of variance (ANOVA) and the chi-square test were used to analyze the data, and the results were considered statistically significant at *p* < 0.05. Excel statistical software (version 2019, Redmond, WA, USA) was used to analyze the data, and GraphPad Prism (version 9.0, San Diego, CA, USA) was used for graphing. SnapGene was used for SNP sequence alignment to determine the genotyping results.

## 3. Results

### 3.1. Association Analysis of SNPs with Hypoxia Tolerance Traits

The rate of oxygen consumption in fish serves as an indicator of the volume of oxygen utilized over a specific time frame, which is intricately linked to their metabolic processes and environmental conditions. In a prior investigation, we identified 52 SNP loci associated with the rate of oxygen consumption (unpublished data). However, the relationship between the oxygen consumption rate and hypoxia tolerance is not straightforward, and it remains unclear whether these loci are associated with hypoxia tolerance. To assess the validity of these SNPs, genotyping was performed on both hypoxia-intolerant and hypoxia-tolerant populations. Among the 52 SNP loci, one was found to be statistically significant, as evidenced by a *p*-value of less than 0.05 (Table 1).

### 3.2. Genotypes Evaluated for Hypoxia Tolerance

**Figure 1:**
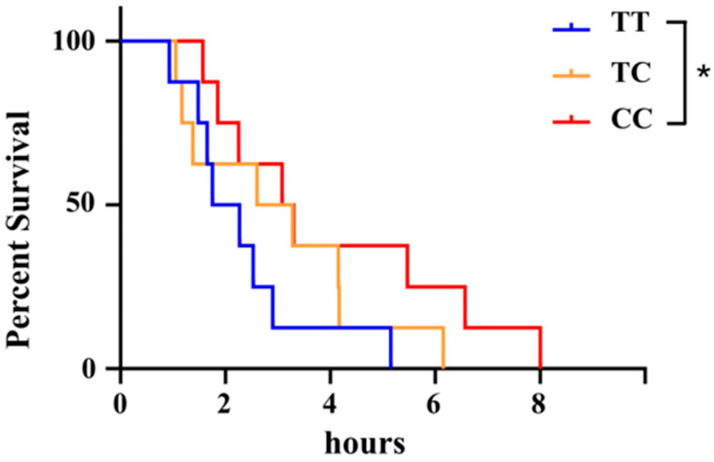
Survival curves of individuals with the TT (blue), TC (orange), and CC (red) genotypes (*n* = 20) under hypoxic stress. There was a statistically significant difference between the TT genotype and TC genotype. * *p* < 0.05.

The three genotypes, TT, TC, and CC, at the Chr8: 29647765 locus, were subjected to hypoxic stress through a process of natural oxygen depletion. The results revealed that under identical experimental conditions and duration, the survival rate of individuals with the CC genotype was significantly higher than that of those with the TT genotype (*p* < 0.05). This finding suggests that the CC genotype confers a distinct advantage, as evidenced by the greater hypoxia tolerance observed in the CC genotype population compared with the TC and TT genotype populations (Figure 1).

### 3.3. Changes in Blood Indices and Oxidative Stress Enzyme Activity by Hypoxia

**Figure 2:**
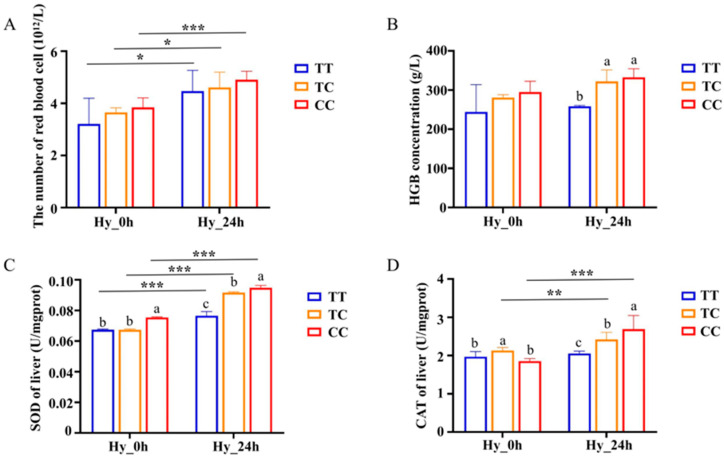
Alterations in red blood cell number (**A**), hemoglobin concentration (**B**), SOD activity (**C**), and CAT activity (**D**) induced by hypoxic conditions across individuals of three different genotypes. Data are shown as the mean ± SD (*n* = 3). Hy_0h refers to normoxia, indicating no exposure to hypoxia. Hy_24h denotes exposure to hypoxia (2 mg/L) for a duration of 24 h. Blue represents the TT genotype, orange represents the TC genotype, and red represents the CC genotype. Distinct letters denote statistically significant differences (*p* < 0.05) among the three genotypes at a single time point. Asterisks indicate statistically significant differences within the same genotype across two time points (* *p* < 0.05, ** *p* < 0.01, *** *p* < 0.001).

Erythrocyte count and hemoglobin concentration were elevated in the Changfeng silver carp after hypoxia treatment. The erythrocyte counts of individuals with the TT, TC, and CC genotypes in normoxia were 2.68 × 10^12^ L^−1^, 3.65 × 10^12^ L^−1^, and 3.84 × 10^12^ L^−1^, respectively. Following 24 h of hypoxic stress, these counts increased to 4.47 × 10^12^ L^−1^, 4.61 × 10^12^ L^−1^, and 4.91 × 10^12^ L^−1^, respectively (Figure 2A). The hemoglobin concentrations for individuals with the TT, TC, and CC genotypes during normoxia were 224.00 g/L, 280.67 g/L, and 294.75 g/L, respectively. After 24 h of hypoxic stress, the hemoglobin concentrations were measured for the TT, TC, and CC genotypes, yielding values of 258.40 g/L, 322.00 g/L, and 332.33 g/L, respectively. Notably, the hemoglobin concentration for the CC and TC genotypes was significantly higher than that of the TT genotype (*p* < 0.05) (Figure 2B). Furthermore, the alterations in antioxidant enzyme activity under hypoxic stress were assessed in individuals possessing the TT, TC, and CC genotypes. The activities of SOD and CAT in serum exhibited an increase with the duration of hypoxic stress across the three distinct genotypes. Of note, after 24 h of hypoxic stress, the antioxidant enzyme activities in the CC genotype were significantly higher compared with those in the TT and TC genotypes (*p* < 0.05) (Figure 2C,D).

### 3.4. Structural Changes in Gill Induced by Hypoxia

**Figure 3:**
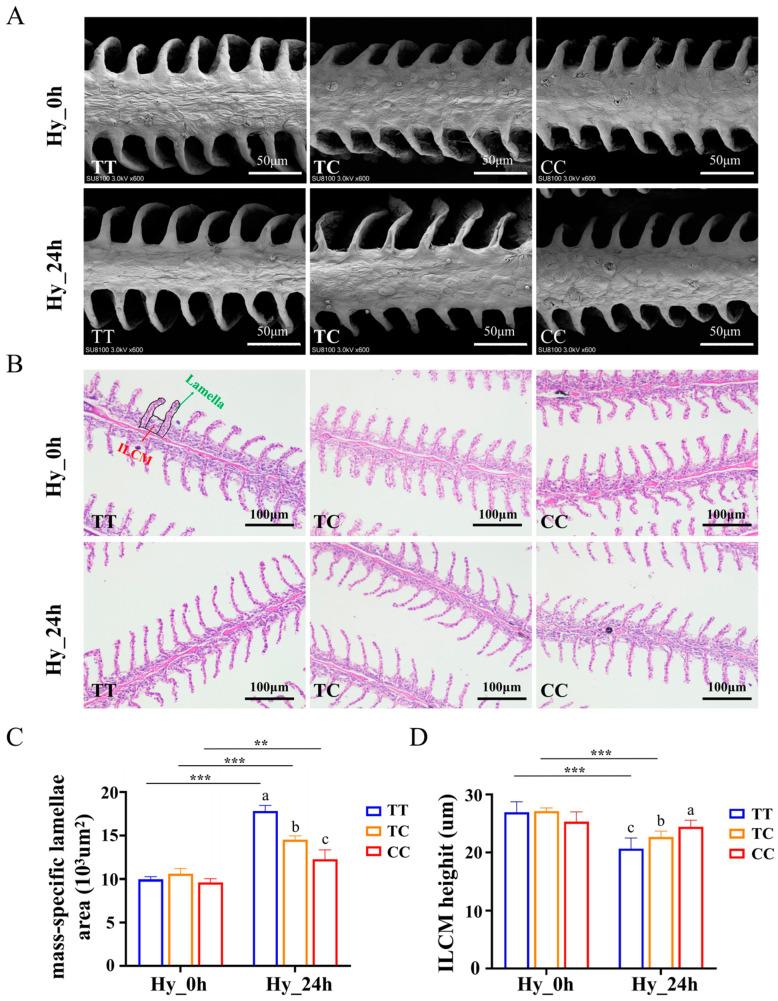
Structural changes in gills induced by hypoxic conditions across the three distinct genotypes. The microstructural analysis of the gills from individuals of various genotypes was conducted using scanning electron microscopy (**A**) and HE staining (**B**), with scale bars located in the lower right-hand corners. (**C**) The surface area measurements of the gill lamellae in individuals of different genotypes following normoxic and hypoxic treatments. (**D**) The variations in interlamellar cell mass (ILCM) thickness among individuals of different genotypes under normoxic and hypoxic conditions. Green arrows denote the gill lamellae, while red arrows indicate the ILCM between two adjacent gill lamellae. Data are shown as the mean ± SD (*n* = 3). Hy_0h refers to normoxia, indicating no exposure to hypoxia. Hy_24h denotes exposure to hypoxia (2 mg/L) for a duration of 24 h. Blue represents the TT genotype, orange represents the TC genotype, and red represents the CC genotype. Distinct letters denote statistically significant differences (*p* < 0.05) among the three genotypes under hypoxic stress. Asterisks indicate statistically significant differences within the same genotype across two time points (** *p* < 0.01, *** *p* < 0.001).

Electron microscopy and HE staining revealed no significant differences in the gill vignette area or the thickness of the interlamellar matrix across the three genotypes under normoxic conditions. However, following 24 h of hypoxic stress, an increase in the surface area of the gill lamellae and a reduction in the thickness of the interlamellar matrix were observed in all three genotypes (Figure 3A,B). The statistical analysis of HE staining showed that under normoxic conditions, the gill lamellae area of the TT genotype measured 9957.54 μm^2^, while that of the CC genotype measured 9619.69 μm^2^. Following 24 h of hypoxic treatment, the gill lamellae area of the TT genotype increased to 17,820.20 μm^2^, whereas the CC genotype exhibited an area of 12,255.14 μm^2^. The gill lamellae area of the CC genotype was significantly larger than that of the TT genotype (Figure 3C) (*p* < 0.05). Under normoxic conditions, the thickness of the interlamellar cell mass (ILCM) between two adjacent gill lamellae measured 26.93 μm for the TT genotype and 25.31 μm for the CC genotype. Following 24 h of hypoxia treatment, these measurements decreased to 20.66 μm for the TT genotype and 24.41 μm for the CC genotype. The interlamellar matrix thickness in the CC genotype gill lamellae was significantly less compared with that in the TT genotype individuals (Figure 3D, *p* < 0.05). As the surface area of the gill filaments increased, there was a corresponding increase in the thickness of the interlamellar matrix, which enhanced the area available for oxygen exchange with the surrounding water. Following hypoxia treatment, the TT genotype exhibited significantly more pronounced structural alterations compared with the CC genotype (*p* < 0.05). Previous research has demonstrated that fish enhance the surface area of their gill respiratory structures and possess the capability for gill remodeling in response to fluctuations in dissolved oxygen levels. These findings indicate that the CC genotype preserves greater structural integrity under hypoxic conditions and demonstrates a superior potentiality to cope with hypoxic stress.

### 3.5. Analysis of Brain Cell Apoptosis Under Hypoxic Stress

**Figure 4:**
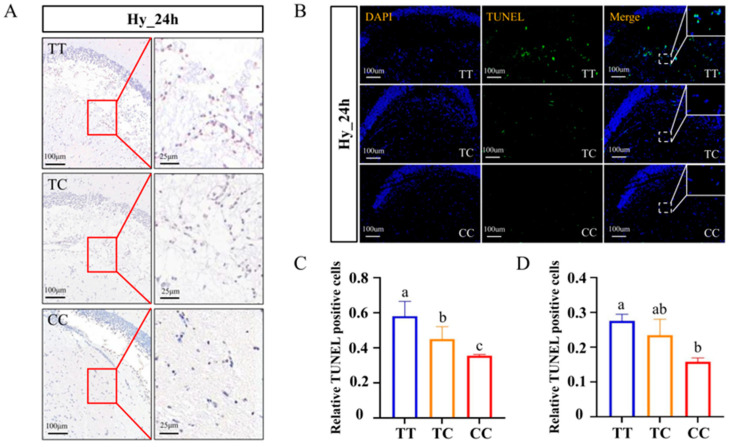
TUNEL staining of the brains in the three genotypes after exposure to hypoxia. (**A**) Light microscope micrographs depict brain cell apoptosis after 24 h of hypoxia. Apoptotic cells are indicated by a brown coloration, whereas normal cells are represented by a light blue hue. The right panel presents a magnified view of the area enclosed within the red box on the left. (**B**) Fluorescence microscopy detection of brain cell apoptosis after 24 h under the hypoxic condition. Apoptotic cells are shown in green fluorescence, DAPI staining (blue) represents the nuclei. The white box located in the upper right corner represents an enlarged view of the area delineated by the dotted line. (**C**) The statistics of the apoptosis rate in the brain under a light microscope after 24 h of hypoxia. (**D**) The statistics of the apoptosis rate in the brain under fluorescence microscopy after 24 h of hypoxia. Data are shown as the mean ± SD (*n* = 3). Blue represents the TT genotype, orange represents the TC genotype, and red represents the CC genotype. Different letters indicate significant differences (*p* < 0.05).

To investigate the underlying causes of varying hypoxia tolerance among different genotypes of silver carp, TUNEL staining was employed to assess cellular apoptosis in the brains of Changfeng silver carp with the TT, TC, and CC genotypes. Under normoxic conditions, the three genotypes exhibited no significant differences in the number of apoptotic cells (Figure S2). However, a marked increase in the number of apoptotic cells in the brain was observed following 24 h of hypoxia exposure, as evidenced by light microscopy (Figure 4A) and fluorescence microscopy (Figure 4B). Statistical analysis revealed a significantly greater number of apoptotic cells in the brains of individuals with the TT genotype compared with those with the TC and CC genotypes following hypoxia treatment (*p* < 0.05) (Figure 4C,D). The observed variation in the number of apoptotic cells between the two detection methods may be attributed to differences in assay sensitivity. These findings elucidate why the TT genotype exhibited a loss of homeostasis at an earlier stage under identical hypoxic conditions, aligning with the increased number of apoptotic cells observed in the brain.

### 3.6. The Characteristics of CTSC Gene and Its Expression Profile

**Figure 5:**
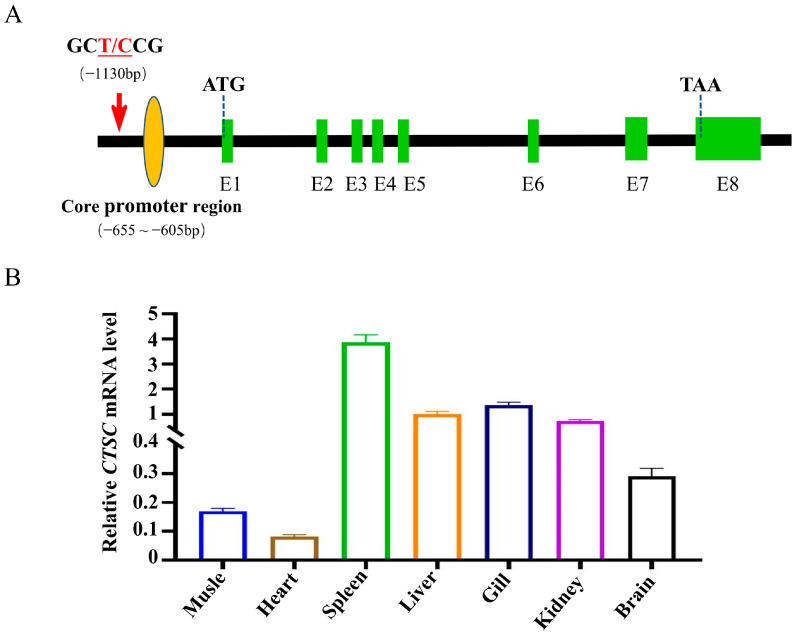
Structural and expression characteristics of the *CTSC* gene. (**A**) Schematic representation of the *CTSC* gene architecture. Orange ovals represent core promoter regions, and green boxes denote exons. (**B**) Expression profiles of the *CTSC* gene across different tissues in silver carp. Data are shown as the mean ± SD (*n* = 3).

Following comprehensive SNP annotation, a promising candidate gene was identified through systematic genomic scanning of the 10 Kb flanking regions upstream and downstream of the significant SNP locus. The *CTSC* gene is situated on chromosome 8 of the silver carp, spanning the genomic coordinates 829637713 to 29646638 and encompasses a complete sequence length of 8926 bp, encompassing eight exons and an open reading frame (ORF) that encodes a polypeptide of 420 amino acids. This gene encodes a protein that is a member of the peptidase C1 family and lysosomal cysteine proteases, which are pivotal in orchestrating the activation of various serine proteases within immune system cells. The core promoter region of the *CTSC* gene is predicted to reside 605 to 655 bp upstream of the ATG initiation codon (Figure 5A). The SNP (Chr8: 29647765; T/C), which is linked to hypoxia tolerance, is positioned 1130 base pairs upstream of the *CTSC* gene. Individuals were randomly selected without consideration of the SNP genotypes to analyze the tissue-specific expression pattern of the *CTSC* gene in silver carp. The results indicate that the *CTSC* gene is expressed across various tissues, with the highest expression levels observed in the spleen, followed by the liver, gill, and kidney. The heart exhibited the lowest expression level (Figure 5B).

### 3.7. Expressional Analysis of CTSC Gene Under Hypoxic Condition

**Figure 6:**
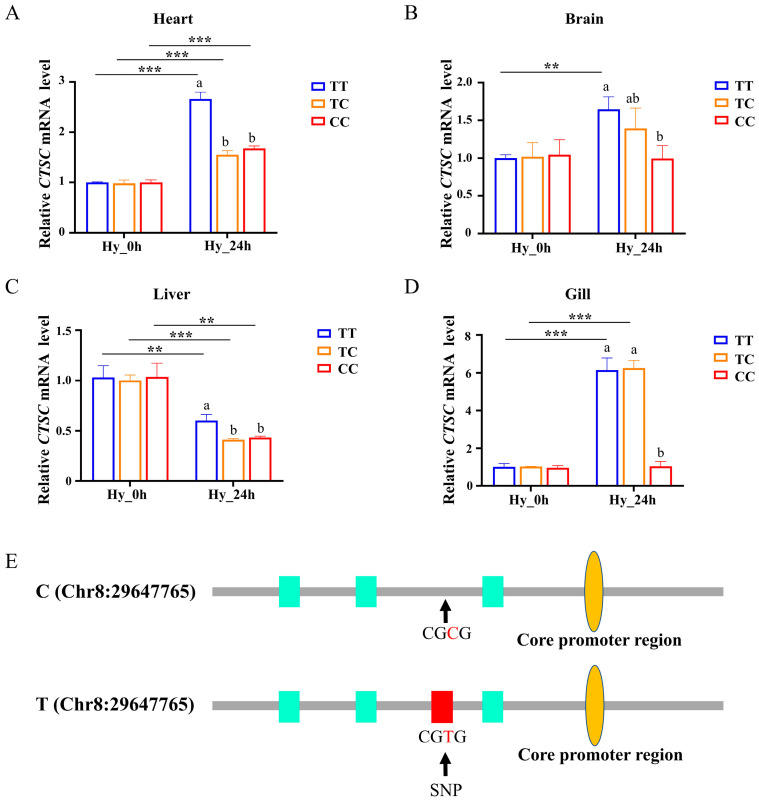
The expression analysis of the *CTSC* gene among the three genotypes under hypoxia stress. Changes in *CTSC* gene expression in the heart (**A**), brain (**B**), liver (**C**), and gill (**D**) in individuals exhibiting three distinct genotypes under both normoxic and hypoxic conditions. (**E**) The prediction of HIF-1α transcription factor binding sites in the upstream region of the core promoter of the *CTSC* gene. Hy_0h refers to normoxia, indicating no exposure to hypoxia. Data are shown as the mean ± SD (*n* = 3). Hy_24h denotes exposure to hypoxia (2 mg/L) for a duration of 24 h. Blue represents the TT genotype, orange represents the TC genotype, and red represents the CC genotype. Cyan boxes represent ACGTG sequences, red boxes represent CGTG sequences, and orange ovals represent core promoter regions. Distinct letters denote statistically significant differences (*p* < 0.05) among the three genotypes under hypoxic stress. Asterisks indicate statistically significant differences within the same genotype across two time points (** *p* < 0.01, *** *p* < 0.001).

The expression of the *CTSC* gene across the heart, brain, liver, and gill tissues in Changfeng silver carp with the TT, TC, and CC genotypes was analyzed. Under normoxic conditions, no significant difference in *CTSC* expression was observed among the three genotypes. After a 24-h exposure to hypoxia at a DO concentration of 2 mg/L, there was an observed increase in the expression of *CTSC* in the heart. Notably, the expression levels in the TT genotype were significantly higher compared with those in the CC genotype (*p* < 0.05) (Figure 6A). In the brain, the *CTSC* gene expression was elevated in the TT genotype, while it remained relatively unchanged in the TC and CC genotypes (Figure 6B). The expression of the *CTSC* gene was downregulated in the liver, with a significantly lower expression observed in the CC genotype compared with other genotypes (*p* < 0.05) (Figure 6C). In contrast, in the gill, the expression of the *CTSC* gene was upregulated in the TT and TC genotypes, while no significant change was detected in the CC genotype. The expression levels in the TT and TC genotypes were significantly higher than those in the CC genotype (*p* < 0.05) (Figure 6D).

Overall, under hypoxic stress, the expression of the *CTSC* gene in the TT and TC genotypes were found to be upregulated in the brain, heart, and gill tissues, while the expression of the *CTSC* gene in the liver was downregulated. These findings are largely consistent with the experimental results observed in randomly selected individuals, irrespective of genotype, subjected to hypoxic stress (Figure S3). Furthermore, following hypoxic stress, the expression level of the *CTSC* gene in individuals with the CC genotype demonstrated upregulation exclusively in the heart, with a rate of increase considerably lower than that observed for the TT genotype. In contrast, expression levels in the brain and gill remained unchanged when compared with the normoxic state. Notably, the expression level in the liver experienced a significant decrease, with the rate of decline markedly exceeding that of the TT genotype. This observation prompted us to hypothesize that the downregulation of the *CTSC* gene may confer an adaptive advantage to organisms in adapting to hypoxic conditions.

HIF-1α is recognized as the principal nuclear transcription factor involved in mediating the hypoxic response. We performed an analysis of the upstream region of the *CTSC* gene core promoter and identified binding sites for the HIF-1α transcription factor (HRE), as illustrated in Figure 6E. Three HIF-1α binding sites (ACGTG) were consistently observed across all three genotypes. Moreover, an additional unique HIF-1α binding site (CGTG) was identified exclusively in individuals possessing an SNP with a T allele located at Chr8: 29647765. Consequently, this binding site sequence (CGTG) was present only in the TT and TC genotypes. These findings imply that the differential expression of the *CTSC* gene in the CC genotype compared with the TT and TC genotypes under hypoxic stress may be attributed to variations in the regulation of HIF-1α.

### 3.8. Analysis of Immune-Related Gene Expression in the Liver After Hypoxia

**Figure 7:**
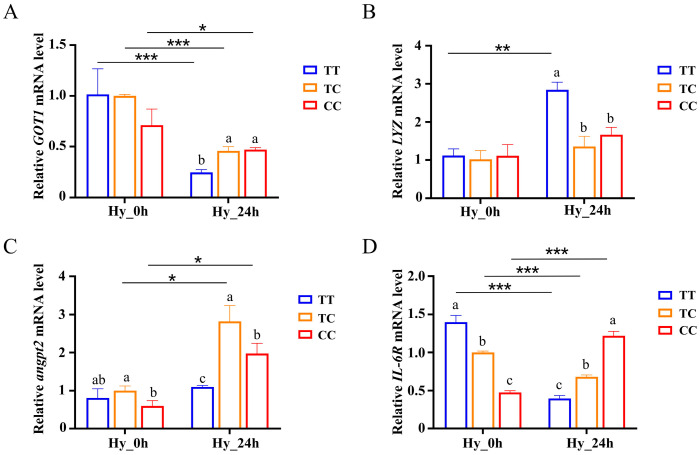
Analysis of immune-related gene expression in the liver after hypoxia. Changes in *GOT1* (**A**), *LYZ* (**B**), *angpt2* (**C**), and *IL-6R* (**D**) gene expression under hypoxia stress. Data are shown as the mean ± SD (*n* = 3). Hy_0h refers to normoxia, indicating no exposure to hypoxia. Hy_24h denotes exposure to hypoxia (2 mg/L) for a duration of 24 h. Blue represents the TT genotype, orange represents the TC genotype, and red represents the CC genotype. Distinct letters denote statistically significant differences (*p* < 0.05) among the three genotypes under hypoxic stress. Asterisks indicate statistically significant differences within the same genotype across two time points (* *p* < 0.05, ** *p* < 0.01, *** *p* < 0.001).

Under normoxic conditions, we assessed the expression levels of enzyme-activated and apoptotic genes across the three genotypes and observed no significant differences in their expression within the liver tissues. However, following hypoxia treatment, there was an upregulation of *Cu/Zn-SOD*, *CAT*, *Caspase3*, and *Caspase9* expression. Notably, a statistically significant difference was identified between the TT and CC genotypes (*p* < 0.05) (Figure S4). Hypoxia causes fish to produce excessive ROS, which induces cellular damage, impairs the immune system, and activates cellular inflammation. To verify whether the alteration of SNPs on the *CTSC* promoter affected the ability of fish to mount an immune response, we analyzed the expression of immune-related genes in the three genotypes. At normoxia, there was no difference in the expression of the *GOT1* and *LYZ* genes among the three genotypes, and the expression of the *angpt2* and *IL-6R* genes was lower in the CC genotype than in the TT genotype. After hypoxia treatment, the gene expression of *GOT1* was decreased, and there was a significant difference between the TT genotype and CC genotype (*p* < 0.05) (Figure 7A). The expression of the *LYZ* gene was sharply increased in the TT genotype and significantly higher than that in the CC genotype (Figure 7B). The *angpt2* gene was increased in the CC genotype (Figure 7C); however, the expression of the *IL-6R* gene was decreased in the TT genotype and increased in the CC genotype (Figure 7D).

### 3.9. Changes of Hepatic Biochemical Indices Induced by Hypoxia

**Figure 8:**
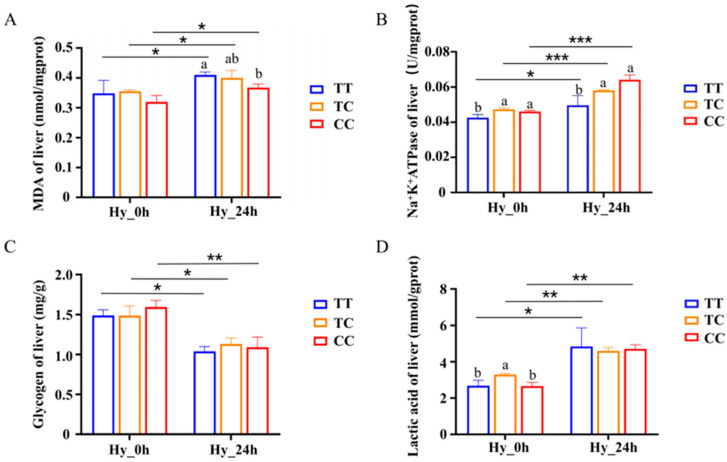
Change in MDA (**A**), Na^+^/K^+^-ATP (**B**), glycogen (**C**), and lactate (**D**) concentration under hypoxia stress. Data are the mean ± SD (*n* = 3). Hy_0h refers to normoxia, indicating no exposure to hypoxia. Hy_24h denotes exposure to hypoxia (2 mg/L) for a duration of 24 h. Blue represents the TT genotype, orange represents the TC genotype, and red represents the CC genotype. Distinct letters denote statistically significant differences (*p* < 0.05) among the three genotypes under hypoxic stress. Asterisks indicate statistically significant differences within the same genotype across two time points (* *p* < 0.05, ** *p* < 0.01, *** *p* < 0.001).

The oxidative stress injury indicator, malondialdehyde (MDA), was tested and found to be elevated after hypoxic treatment. Individuals with the CC genotype demonstrated significantly lower MDA levels compared with those with the TT genotype (*p* < 0.05) (Figure 8A). Moreover, hepatic energy indices were assessed following a 24-h exposure to hypoxia across the three different genotypes. Following 24 h of hypoxia, the hepatic Na^+^/K^+^-ATPase content exhibited an increase, with a statistically significant elevation in Na^+^/K^+^-ATPase levels observed in individuals possessing the CC genotype compared with those with the TT genotype (*p* < 0.05) (Figure 8B). Concurrently, the lactate levels rose post-hypoxia treatment; however, no statistically significant differences were detected in lactate content across the three genotypes (Figure 8B). Additionally, the glycogen content demonstrated a decrease, with no significant variation observed among the three genotypes (Figure 8C).

## 4. Discussion

Silver carp are less tolerant to low oxygen concentration in the water column, which can compromise the immune system and lead to reduced resistance to pathogenic infections, potentially causing severe economic losses to the aquaculture industry. Hypoxia tolerance in fish is one of the heritable quantitative traits that is controlled by multiple genes. Recently, researchers have made progress in fish selection for growth and hypoxia tolerance. The Changfeng silver carp is a new variety characterized by stable inheritable traits, rapid growth, and tolerance to low oxygen levels. In this study, we screened important hypoxia-tolerant SNP loci using GWAS data and validated their dominant genotypes. This SNP locus can serve as a molecular characterization marker for new hypoxia-tolerant silver carp lines and can be used in the molecular-assisted breeding of silver carp.

The survival rate serves as a crucial indicator of a fish’s capacity to withstand low oxygen levels. Under identical DO concentrations, fish that exhibited lateral flipping at a later stage demonstrated an enhanced ability to adapt to reduced DO levels. In this study, fish possessing the TT genotype exhibited a loss of equilibrium and sank to the bottom of the water more rapidly than those with the CC genotype, suggesting that the CC genotype confers greater tolerance to the hypoxic condition (Figure 1). These findings suggest that the CC genotype may possess a superior capacity for acclimatization to low-oxygen environments, thereby prolonging the survival time.

Hematological indices of fish reflect their physiological status and health, and hypoxic stress significantly affects their blood composition [26,27]. The oxygen-carrying capacity of the blood is related to the erythrocyte count, and fish respond to hypoxia by increasing their erythrocyte count. In this study, the erythrocyte counts and hemoglobin concentration increased after 24 h of hypoxia (Figure 2A,B). The hemoglobin concentration of fish with the TC and CC genotypes was significantly higher than that of the TT genotype under hypoxic conditions, indicating that fish with the TC and CC genotypes exhibit higher hemoglobin oxygen affinity, improving their oxygen-carrying capacity. Compared with hemoglobin, erythrocyte counts did not exhibit significant alterations during hypoxic conditions. This phenomenon may be attributed to the greater responsiveness of hemoglobin, an oxygen transport molecule, to hypoxia. Under hypoxic conditions, both the concentration and transport rate of hemoglobin increase to sustain adequate oxygenation in the body. In contrast, the extent of change in erythrocyte levels is less pronounced compared with that of hemoglobin [28].

Oxidative stress stimulates organisms to activate antioxidant defense mechanisms, thereby maintaining dynamic homeostasis and improving resistance in fish [29]. In the antioxidant system, the earliest changes occur in a series of antioxidant enzymatic reactions. Several studies have demonstrated that under hypoxic conditions, the levels of superoxide dismutase (SOD) and catalase (CAT) in largemouth bass increase [30]. This phenomenon has also been observed in *Megalobrama megalobrama* [22]. SOD and CAT are important antioxidant enzymes that can scavenge superoxide radicals (O^−2^) and peroxides in the body and protect organisms from oxidative damage. In our study, the activities of antioxidant enzymes, including catalase (CAT) and superoxide dismutase (SOD), exhibited significant variation among the three genotypes under hypoxia stress (*p* < 0.05) (Figure 2C,D). The higher levels of SOD and CAT observed in the CC genotype suggest a potential adaptive mechanism employed by the organism to safeguard tissues against hypoxic damage. In hypoxic conditions, fish modulate their antioxidant system by increasing the activity of various antioxidant enzymes, thereby mitigating or maintaining oxidative stress within a controlled range [31].

Gills are the main respiratory organ of fish, and they adapt to the hypoxic environment by increasing the respiratory surface area of the gills under hypoxic conditions [9,32]. Our study revealed that the respiratory surface area of the gill lamellae of individuals with the TT, TC, and CC genotypes increased, and the volume of the interlayer matrix decreased under hypoxic conditions. After 24 h of hypoxia, the gill lamellae extension height and respiratory area were significantly smaller in fish with the CC genotype than in the TT and TC genotypes (Figure 3). These data indicate that CC genotype fish have more intact gill structures under hypoxic conditions, indicating a higher tolerance to hypoxia, which may have a protective effect on the gill structure. Similar results have been obtained in zebrafish, where usp3-deficient purebreds exhibited more intact gill filaments and higher hypoxia tolerance [33].

Hypoxia stimulates an elevated production of reactive oxygen species (ROS) within the brain, culminating in alterations to cellular structure and function, ultimately resulting in oxidative damage, apoptosis, and the necrosis of neurons [34]. In the current study, our findings indicate that the prevalence of apoptotic cells in the brain was significantly reduced in individuals with the CC genotype compared with those with the TT genotype (Figure 4). Oxidative stress serves as a potent trigger for apoptosis, leading to neuronal cell death under hypoxic conditions [35]. The reduced level of oxidative stress observed in the CC genotype can be attributed to the elevated content of superoxide dismutase (SOD) and catalase (CAT) enzymes (Figure 2C,D), resulting in a decreased number of apoptotic cells in the brains of individuals with this genotype. Selective breeding for specific genotypes at various loci may not only enhance the hypoxia-tolerance traits in silver carp, but also facilitate the development of novel hypoxia-tolerant strains.

*CTSC* is a class of protein hydrolases widely found in the lysosomes of many tissues and plays a crucial role in various physiological activities in the body. As one of the most important protein hydrolases, the tissue-specific expression of *CTSC* has been extensively studied in mammals and other aquatic species [36,37,38,39]. *CTSC* is abundant in the liver, spleen, and kidney of mice but is very low in the heart and brain [40]. Among the various tissues of the golden pomfret, *CTSC* expression was the highest in the muscle, followed by the liver and head kidney, with relatively low expression in the stomach, skin, and brain [41]. Similar to the distribution patterns in mammals, the results revealed that *CTSC* was widely distributed in various tissues of silver carp, with high expression levels in the spleen, kidney, and liver and low expression levels in the heart and muscle (Figure 5B). The relatively high expression of *CTSC* in some immune tissues indicates that it may have a specific immune function.

Through association analysis of the SNP loci, it was determined that the SNP was situated in the upstream promoter region of the *CTSC* gene (Figure 5A). We hypothesized that variations in the genotypes of these SNP loci might influence the expression of the *CTSC* gene. Consequently, we examined the expression levels of the *CTSC* gene across three genotypes under conditions of hypoxic stress. The liver, brain, heart, and gills are critical tissues sensitive to hypoxia and are closely associated with energy and oxygen metabolism. Therefore, we selected these four tissues to assess the expression of *CTSC* across different genotypes following 24 h of hypoxic stress.

Our findings indicate that the expression of the *CTSC* gene was significantly reduced in the CC genotype compared with the TT genotype under hypoxic conditions (Figure 6A–D). This reduced expression may facilitate the improved adaptation of organisms to hypoxia. Furthermore, analysis of the DNA sequences upstream of the *CTSC* gene revealed the presence of three hypoxia-responsive element (HRE) sequences in the C genotype and four in the T genotype (Figure 6E). Variations in allelic single nucleotide polymorphisms (SNPs) result in differing numbers of hypoxia-inducible factor 1-alpha (HIF-1α) binding sites across genotypes. Following 24 h of hypoxic conditions, the expression levels of the CC genotype were observed to be lower compared with those of the TT and TC genotypes, likely as a result of regulation by HIF-1α. Based on the aforementioned results, it is hypothesized that the enhanced hypoxia tolerance observed in the CC genotype may be due to its lower expression level of the CTSC gene than the other two genotypes, and that this reduced expression level may enhance hypoxia tolerance by affecting the HIF-1α signaling pathway.

Gene expression analysis across various tissues under hypoxic stress revealed that the liver was the sole tissue exhibiting downregulation of *CTSC* expression. Consequently, we propose that *CTSC* may play a critical role in the liver’s adaptation to hypoxic conditions. Studies have shown that *CTSC* is essential for antimicrobial immune responses in mammals. In aquatic animals, in vitro overexpression of *CTSC* from Epinephelus coioides significantly delayed the SGIV-induced cytopathic effect (CPE) process and inhibited viral gene transcription [42]. Hypoxia elevates the intracellular levels of reactive oxygen species (ROS), inducing oxidative stress that can potentially compromise immune cell integrity [43]. This oxidative damage may impair the functionality of key fish immune cells, including macrophages and lymphocytes, thereby diminishing their pathogen-clearing capacity [10]. In the present study, we identified specific single nucleotide polymorphism (SNP) sites within the *CTSC* gene that modulate hypoxia tolerance and immune response in fish. Notably, individuals with the CC genotype demonstrated an elevated expression of antioxidant enzymes (Cu/Zn-SOD and CAT) under hypoxic stress, reflecting enhanced antioxidant defense mechanisms that effectively mitigated immune cell damage. In contrast, the TT genotype was associated with increased caspase-3 activity under hypoxic conditions, suggesting heightened apoptotic activity. Furthermore, hypoxia exposure triggered the rapid upregulation of immune-related genes (*GOT1*, *LYZ*, *IL-6R*, and *angpt2*) in the CC genotype, indicating a more pronounced and efficient immune activation. Under hypoxic conditions, HIF-1α serves as a crucial transcriptional regulator that activates the expression of various genes, particularly those encoding antioxidant enzymes and immune-related factors. The enhanced expression of antioxidant enzymes and the rapid upregulation of immune-related genes observed in the CC genotype are likely mediated through intricate gene regulatory networks and gene–environment interactions. This sophisticated regulatory mechanism indicates that SNP do not exert their phenotypic effects in isolation, but rather through the integrated actions of multiple genetic elements and environmental influences.

MDA is a classical marker of oxidative stress, formed by lipid peroxidation; it can lead to the loss of cell function and DNA damage. The present study showed that after hypoxic stress, the CC genotype had lower levels of oxidative damage and were more adapted to the hypoxic environment (Figure 8A). Na^+^/k^+^-ATPase is a key enzyme involved in energy metabolism, and an increase in Na^+^/K^+^-A TPase activity suggests that more energy is required in response to hypoxia, and that enhancement of Na^+^/K^+^-ATPase activity is essential in the adaptation to acute hypoxia. Aquatic organisms can adapt to hypoxia by regulating their energy metabolism to maintain an adequate energy supply [44]. Hepatic glycogen is an important source of energy during hypoxia and can provide a sufficient glucose substrate for anaerobic metabolism [45,46]. Fish can fulfill their energy requirements by activating the glycolytic pathway under hypoxic stress [47]. Under hypoxic conditions, glucose is converted to pyruvate and the end product is lactate, leading to lactate accumulation [48,49]. After 24 h of hypoxia treatment, the glycogen content decreased and the lactate content increased, which indicated that the organism was undergoing glycolysis, but there was no significant difference between genotypes (Figure 8C,D). The Na^+^/K^+^-ATPase content was elevated and was significantly higher in the CC than in the TT genotype, indicating that the CC genotype was more adapted to the hypoxic environment. It has been found that Na^+^/K^+^-ATPase/Src signaling activation plays an important role in increasing the production of reactive oxygen species (ROS) [50]. In addition, *CTSC* induces a significant increase in ROS levels, and the main sources of intracellular ROS are NADPH oxidase and mitochondria [51], which can affect the activity of Na^+^/K^+^-ATPase by oxidatively modifying it, and thus interfere with cellular electrolyte homeostasis and function, leading to cell injury or death. Therefore, both *CTSC* and Na^+^/K^+^-ATPase affect the ROS content, and we hypothesized that the lower *CTSC* expression under hypoxia may affect the Na^+^/K^+^-ATPase content in the liver.

Although our study identified an association between this SNP and the *CTSC* gene as well as phenotypic variation, it is important to emphasize that this association may result from a combination of mutations, multiple genetic factors, and environmental influences. The effect of a single SNP could be mediated indirectly through complex genetic networks or gene–environment interactions [52]. For instance, the SNP might regulate the expression of other genes or interact with specific environmental factors to influence the phenotype. Furthermore, complex traits are typically polygenic, with each genetic variant contributing modestly to the overall phenotypic variation. Therefore, future studies should integrate multi-omics data and incorporate analyses of environmental factors to more comprehensively elucidate the functional mechanisms of this SNP and its role in phenotypic variation.

## 5. Conclusions

In this study, a significant SNP locus at Chr8: 29647765 (T/C) was identified in Changfeng silver carp, demonstrating an association with hypoxia tolerance traits. The homozygous CC genotype exhibited greater tolerance to hypoxic conditions compared with the homozygous TT genotype. Under hypoxic conditions, the expression levels of the *CTSC* gene were significantly lower in the CC genotype compared with the TT and TC genotypes, suggesting that the SNP linked to the CC genotype resulted in decreased *CTSC* expression. This reduction in expression may contribute to enhanced physiological adaptation to hypoxia. The analysis of the promoter region of *CTSC* identified a distinct predicted hypoxia-inducible factor 1-alpha (HIF-1α) binding site (CGTG) in the T genotype. It was observed that the expression levels of the CC genotype were lower compared with those of the TT and TC genotypes, likely due to regulation by HIF-1α. The expression analysis of genes associated with immunoenzyme activity demonstrated a positive correlation between the hypoxia tolerance of the Changfeng silver carp and its immune response under hypoxic conditions. However, when synthesizing these observations, we hypothesized that the observed combination of effects may not only be a direct result of individual SNP, but is more likely to be the result of a combination of multiple genetic and environmental factors. Although single nucleotide polymorphisms may be important influences, their effects may manifest indirectly through complex gene networks or environmental interactions. This study successfully identified a significant SNP site in Changfeng silver carp, offering valuable insights for future selective breeding and the development of novel hypoxia-tolerant varieties.

## Figures and Tables

**Table 1:** A single SNP associated with hypoxia tolerance identified by the chi-square test in two silver carp populations.

SNP Site	Genotypes	Number of Hypoxia-Tolerant Individuals	Number of Hypoxia-Intolerant Individuals	X^2^	*p*-Value	Gene Annotation	Position
Chr8: 29647765	TT	29	26	8.001	0.018	Cathepsin C (*CTSC*)	1130 bp upstream
	CC	16	34				
	TC	68	55				

## Data Availability

The data presented in this study are available in the article.

## Supplementary Materials

The following supporting information can be downloaded at: https://www.mdpi.com/article/10.3390/ani15040532/s1, Figure S1: A brief diagram of the hypoxia stress experiment. Figure S2: TUNEL staining of brains of the three genotypes under normoxia. Figure S3: Changes in CTSC gene expression in the heart tissue (A), brain tissue (B), liver tissue (C), and gill tissue (D) under hypoxic conditions. Figure S4: Effects of hypoxia treatment on the expression of immunoenzyme genes and apoptosis genes in Changfeng silver carp. Table S1: The primer sequences used in this study. Table S2: The assay kits used in this study.
