# Supplementary material for: A 5′ Promoter Region SNP in CTSC Leads to Increased Hypoxia Tolerance in Changfeng Silver Carp (Hypophthalmichthys molitrix)
Source: Animals (Basel). 2025 Feb 13;15(4):532. doi: 10.3390/ani15040532 (PMC11851654; doi:10.3390/ani15040532)
Supplement: Supplementary file 1 [file animals-15-00532-s001.zip › Figure S2.pdf]

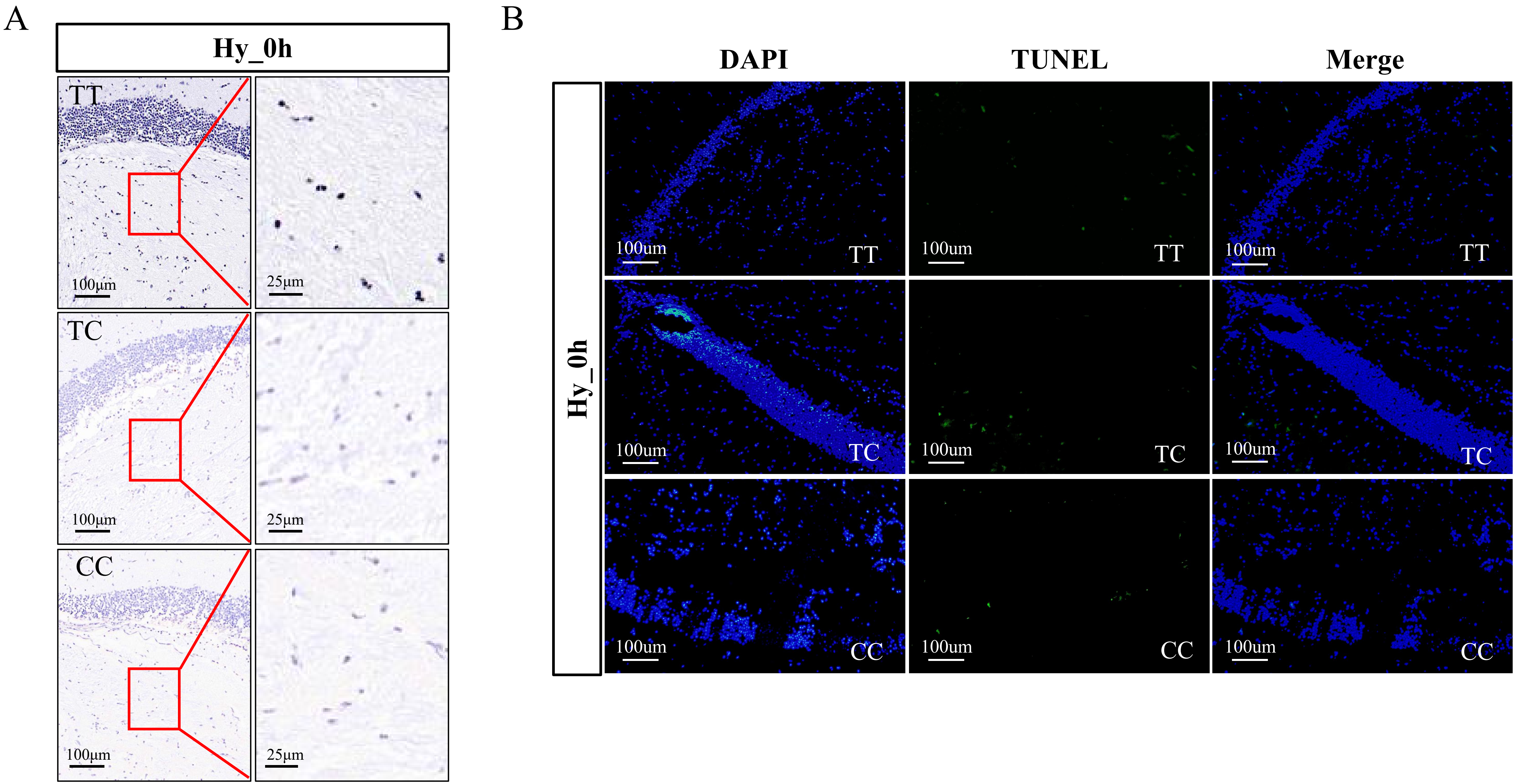

Figure S2. TUNEL staining of brains of three genotypes under normoxia. (A) Light microscope micrograph showing apoptotic brain cells under normoxia. Apoptotic cells are brown in color and normal cells are light blue. On the right is a magnified view of the red boxed portion on the left. (B) Fluorescence microscopy to detect apoptosis of brain cells under normoxia. Apoptotic cells are shown as green fluorescence and DAPI staining (blue) represents the nucleus.
