# Supplementary material for: A 5′ Promoter Region SNP in CTSC Leads to Increased Hypoxia Tolerance in Changfeng Silver Carp (Hypophthalmichthys molitrix)
Source: Animals (Basel). 2025 Feb 13;15(4):532. doi: 10.3390/ani15040532 (PMC11851654; doi:10.3390/ani15040532)
Supplement: Supplementary file 1 [file animals-15-00532-s001.zip › Figure S3.pdf]

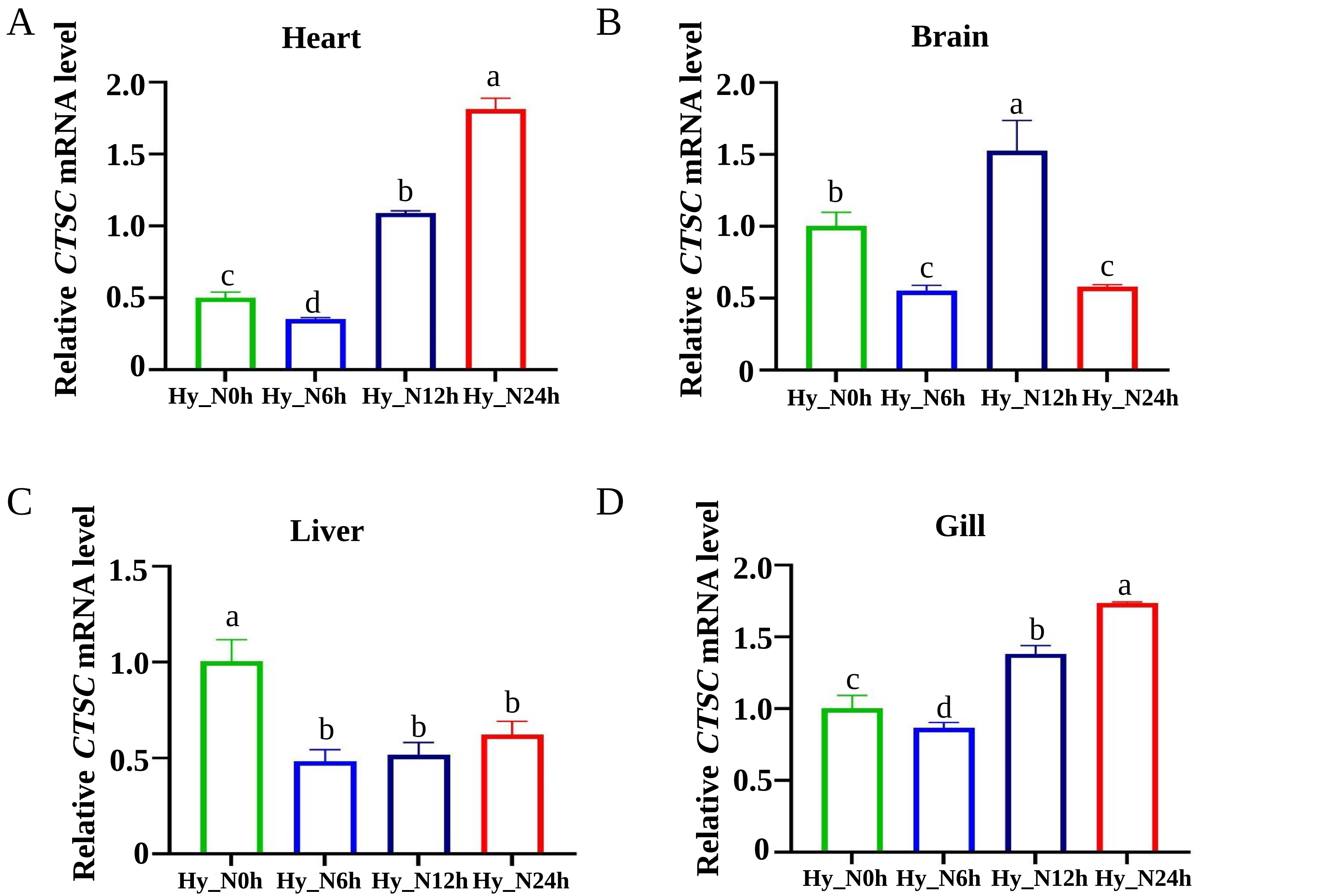

Figure S3. Changes of *CTSC* gene expression in heart tissue (A), brain tissue (B), liver tissue (C) and gill tissue (D) under hypoxic conditions. Distinct letters indicate statistically significant differences ($P < 0.05$). Hy_N0h, refers to normoxia, indicating no exposure to hypoxia. Hy_N6h denotes exposure to hypoxia (2 mg/L) for a duration of 6 hours. Hy_12h denotes exposure to hypoxia (2 mg/L) for a duration of 12 hours. Hy_N24h denotes exposure to hypoxia (2 mg/L) for a duration of 24 hours. Date was shown as mean±SD (n=3).
