# Supplementary material for: A 5′ Promoter Region SNP in CTSC Leads to Increased Hypoxia Tolerance in Changfeng Silver Carp (Hypophthalmichthys molitrix)
Source: Animals (Basel). 2025 Feb 13;15(4):532. doi: 10.3390/ani15040532 (PMC11851654; doi:10.3390/ani15040532)
Supplement: Supplementary file 1 [file animals-15-00532-s001.zip › Figure S4.pdf]

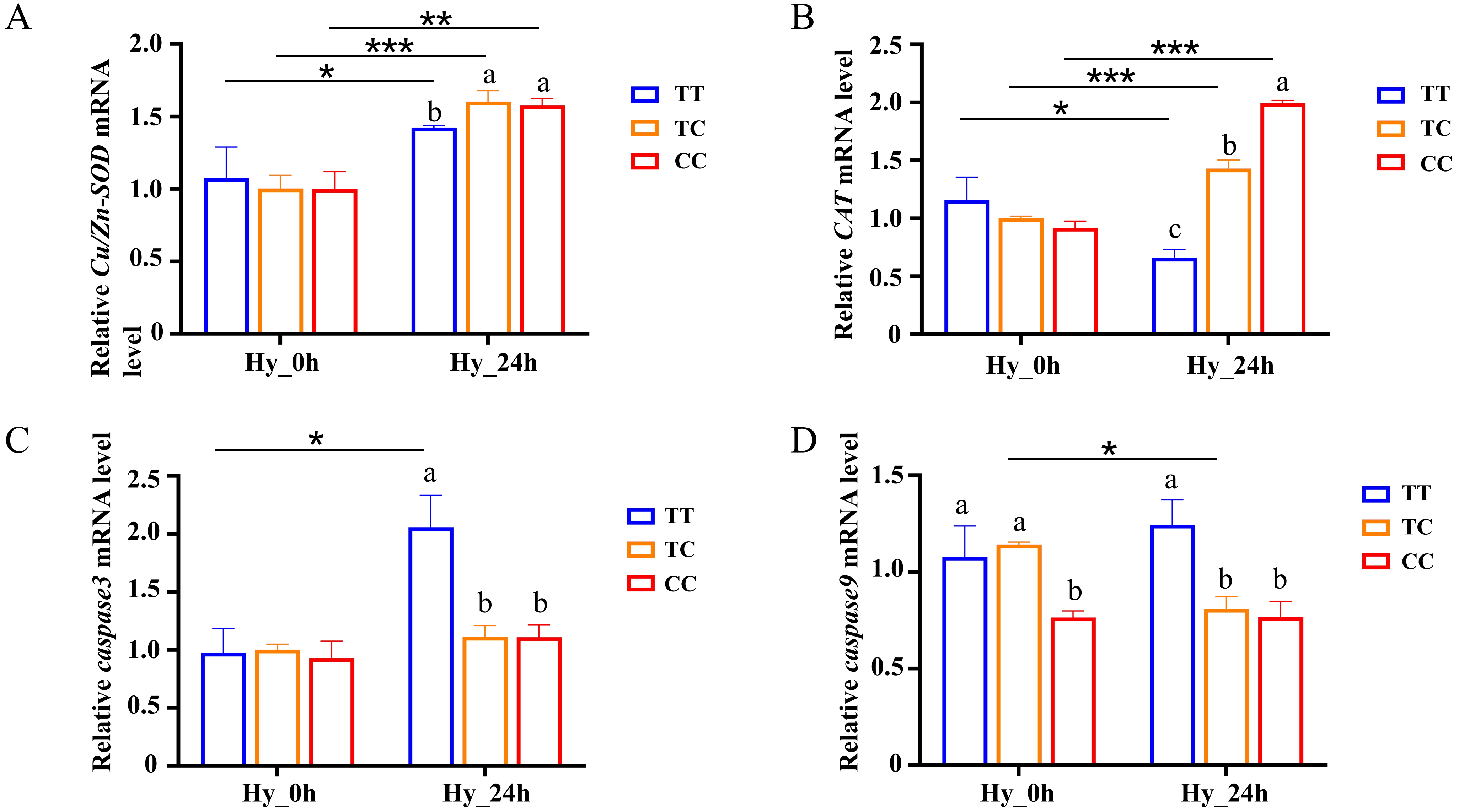

Figure S4. Effects of hypoxia treatment on the expression of immunoenzyme genes and apoptosis genes in Changfeng silver carp. Changes in *Cu/Zn-SOD* (A), *CAT* (B), *caspase3* (C) and *caspase9* (D) gene expression under hypoxia stress. Date was shown as mean±SD (n=3). Hy_0h, refers to normoxia, indicating no exposure to hypoxia. Hy_24h denotes exposure to hypoxia (2 mg/L) for a duration of 24 hours. Blue represents the TT genotype, orange represents the TC genotype, and red represents the CC genotype. Distinct letters denote statistically significant differences ($P < 0.05$) among the three genotypes under hypoxic stress. Asterisks indicate statistically significant differences within the same genotype across two time points ($\ast P < 0.05$, $\ast\ast P < 0.01$, $\ast\ast\ast P < 0.001$).
