# Supplementary material for: A 5′ Promoter Region SNP in CTSC Leads to Increased Hypoxia Tolerance in Changfeng Silver Carp (Hypophthalmichthys molitrix)
Source: Animals (Basel). 2025 Feb 13;15(4):532. doi: 10.3390/ani15040532 (PMC11851654; doi:10.3390/ani15040532)
Supplement: Supplementary file 1 [file animals-15-00532-s001.zip › Table S1.pdf]

Table S1. The primer sequences used in this study.

Primer name	Sequence of primers (5′-3′)	Products length/bp	Annealing temperature/°C	Application
Chr8:29647765-F	GCATCATTACGTCACGTCTG	605	56	For screening of SNP site
Chr8:29647765-R	ACAATGTTGTCGCCTGTACG			
CTSC-F	ACTTCGGCATTGTGGAGGAG	121	60	For RT-qPCR
CTSC-R	CCGTAAAACCCACCCACGTA			
Cu/Zn-SOD-F	ACACGTCGGAGACCTTGGTA	175	60	For RT-qPCR
Cu/Zn-SOD-R	TGCCTATAACACCACAGGCC			
CAT-F	TCGCTGTACGCTTTTCCACT	174	59	For RT-qPCR
CAT-R	TCCGGATCCTTCAGGTGAGT			
GOT1-F	GGAAGCAGATCGCTGATGTC	162	59	For RT-qPCR
GOT1-R	ACAGTCAGGTTCCCCACTCT			
LYZ-F	GAGTCATCATCATGGGCCCT	186	58	For RT-qPCR
LYZ-R	TCCATCTGCTTTGCTCTGCC			
Caspase3-F	ATCTGGACTCTGGCATTGAG	158	56	For RT-qPCR
Caspase3-R	CCTGCATGATCTCCAGTTGC			

Caspase9-F	CAGCCTTCACTGATCCCTCT	175	58	For RT-qPCR
Caspase9-R	CACGGACTGGCATCCATCTT			
IL-6R-F	CGGTCACTATGAAACCCCCT	228	58	For RT-qPCR
IL-6R-R	GGACGTCTTAAAATCCCACC			
angpt2-F	CAGGAAAGAAGCAGTACCAG	220	56	For RT-qPCR
angpt2-R	GTGGTTTTGCACCACGTCAT			
40s ribosomal RNA	CGCAACAACGGCAAGAAACT	144	59	For RT-qPCR
40s ribosomal RNA	ACACGTCAACAGCCTGTCTC			
