# Supplementary material for: A 5′ Promoter Region SNP in CTSC Leads to Increased Hypoxia Tolerance in Changfeng Silver Carp (Hypophthalmichthys molitrix)
Source: Animals (Basel). 2025 Feb 13;15(4):532. doi: 10.3390/ani15040532 (PMC11851654; doi:10.3390/ani15040532)
Supplement: Supplementary file 1 [file animals-15-00532-s001.zip › Table S2.pdf]

Table S2. The assay kits used in this study.

Reagent	Sourse	Identifier
CAT assay Kit	Nanjing Jiancheng Bio.	A007-2-1
SOD assay kit	Nanjing Jiancheng Bio.	A001-1-2
Lactic Acid assay kit	Nanjing Jiancheng Bio.	A019-2-1
Liver / Muscle glycogen assay kit	Nanjing Jiancheng Bio.	A043-1-1
MDA assay kit	Nanjing Jiancheng Bio.	A003-1-2
Na^+/K^+-ATP assay kit	Nanjing Jiancheng Bio.	A070-2-2
