# Supplementary figures and images for: A 5′ Promoter Region SNP in CTSC Leads to Increased Hypoxia Tolerance in Changfeng Silver Carp (Hypophthalmichthys molitrix)
Source: Animals (Basel). 2025 Feb 13;15(4):532. doi: 10.3390/ani15040532 (PMC11851654; doi:10.3390/ani15040532)

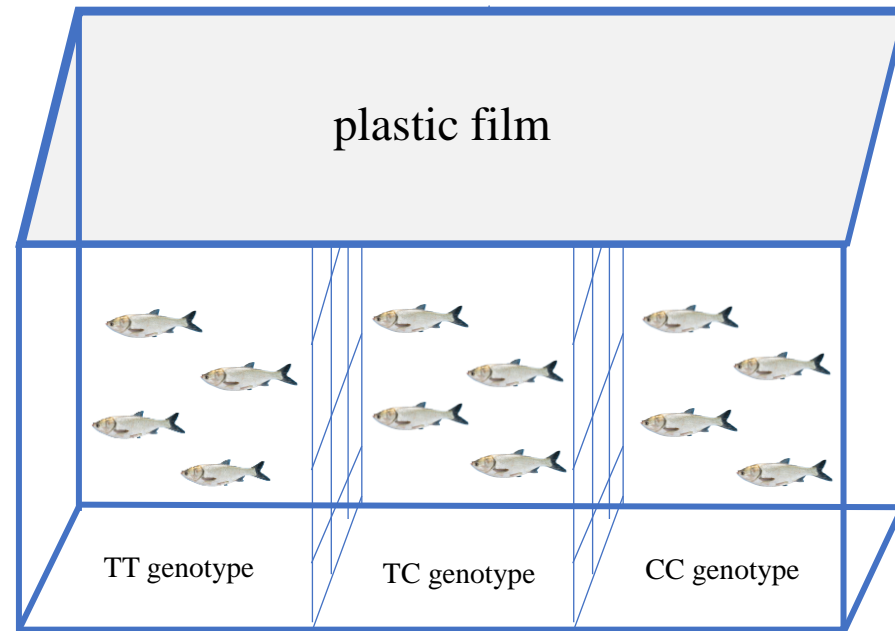

Figure S1. A brief diagram of the hypoxia stress experiment. Gray represents plastic film.

Supplement: Supplementary file 1 [file animals-15-00532-s001.zip › Figure S1.pdf]
